# p53-targeted lincRNA-p21 acts as a tumor suppressor by inhibiting JAK2/STAT3 signaling pathways in head and neck squamous cell carcinoma

**DOI:** 10.1186/s12943-019-0993-3

**Published:** 2019-03-11

**Authors:** Shufang Jin, Xi Yang, Jiayi Li, Wenyi Yang, Hailong Ma, Zhiyuan Zhang

**Affiliations:** 1 Department of Oral Maxillofacial-Head and Neck Oncology, Shanghai Ninth People’s Hospital, College of Stomatology, Shanghai Jiao Tong University School of Medicine, No 639, Zhizaoju Rd, Shanghai, 200011 China; 2 National Clinical Research Center for Oral Diseases, Shanghai, 200011 China; 3 Shanghai Key Laboratory of Stomatology & Shanghai Research Institute of Stomatology, Shanghai, 200011 China

**Keywords:** Long intergenic noncoding RNA p21, p53, NF-YA, Signal transducer and activator of transcription 3, Head and neck squamous cell carcinoma

## Abstract

**Background:**

Long intergenic noncoding RNA p21 (lincRNA-p21) is considered a target of wild-type p53, but little is known about its regulation by mutant p53 and its functions during the progression of head and neck squamous cell carcinoma (HNSCC).

**Methods:**

RNAscope was used to detect the expression and distribution of lincRNA-p21. Chromatin immunoprecipitation and electrophoretic mobility shift assays were performed to analyze the transcriptional regulation of lincRNA-p21 in HNSCC cells. The biological functions of lincRNA-p21 were investigated in vitro and in vivo. RNA immunoprecipitation and pull-down assays were used to detect the direct binding of lincRNA-p21.

**Results:**

Lower lincRNA-p21 expression was observed in HNSCC tissues and indicated worse prognosis. Both wild and mutant type p53 transcriptionally regulated lincRNA-p21, but nuclear transcription factor Y subunit alpha (NF-YA) was essential for mutant p53 in the regulation of lincRNA-p21. Ectopic expression of lincRNA-p21 significantly inhibited cell proliferation capacity in vitro and in vivo and vice versa. Moreover, the overexpression of lincRNA-p21 induced G1 arrest and apoptosis. Knockdown NF-YA expression reversed tumor suppressor activation of lincRNA-p21 in mutant p53 cells, not wild-type p53 cells. A negative correlation was observed between lincRNA-p21 and the phosphorylation of signal transducer and activator of transcription 3 (p-STAT3) in HNSCC tissues. High lincRNA-p21 expression inhibited Janus kinase 2 (JAK2)/STAT3 signal activation and vice versa. Further, we observed direct binding to STAT3 by lincRNA-p21 in HNSCC cells, which suppressed STAT3-induced oncogenic potential.

**Conclusions:**

Our results revealed the transcriptional regulation of lincRNA-p21 by the mutant p53/NF-YA complex in HNSCC. LincRNA-p21 acted as a tumor suppressor in HNSCC progression, which was attributed to direct binding to STAT3 and blocking of JAK2/STAT3 signaling.

**Electronic supplementary material:**

The online version of this article (10.1186/s12943-019-0993-3) contains supplementary material, which is available to authorized users.

## Background

Head and neck squamous cell carcinoma (HNSCC) is an aggressive malignancy with high local recurrence and lymph node metastasis that affects 600,000 new patients worldwide per year [1]. Despite great progress in genomic and molecular research, the treatment effects on patients with HNSCC remains unsatisfactory [2]. Exploration of the complex molecular mechanisms underlying HNSCC occurrence and development is still urgent.

Accumulating studies show that long noncoding RNAs (lncRNAs) participate in diverse physiological and pathological processes, such as cell cycle, apoptosis and metabolism [3–6], as well as reprogramming of induced pluripotent stem cells [7], organ fibrosis [8], and initiation of cancer [9]. HOX transcript antisense RNA (HOTAIR), a well-established lncRNA, is overexpressed in multiple human cancers and acts as a potential prognostic biomarker [10, 11]. HOTAIR interacts with enhancer of zeste homolog 2 to promote the growth of HNSCC cells [12]. Overexpression of lncRNA H19 predicts poor prognosis in colorectal cancer [13], gastric cancer [14], and HNSCC patients [15]. An accumulating number of lncRNAs have been confirmed to play a critical role in cancer progression. Long intergenic noncoding RNA p21 (lincRNA-p21) was first identified as a repressor of the p53-dependent apoptotic response after DNA damage in mouse embryonic fibroblasts harboring wild-type p53 [16]. LincRNA-p21 also provides feedback to enhance wild-type p53 transcriptional activity in vascular smooth muscle cells [17]. Moreover, 85% of HNSCC patients harbor mutations in the *TP53* gene [18, 19]. Mutation of the *TP53* gene can not only result in loss of wild-type p53 function or exert a dominant-negative effect over the remaining wild-type allele but also lead to a gain in oncogenic properties that promote tumor growth [20]. As a transcriptional factor, p53 not only transcribes messenger RNAs but also noncoding RNAs. Whether lincRNA-p21 participates in carcinogenesis and whether its regulation is dependent on p53 status in HNSCC are still unknown.

In this study, we demonstrated that lincRNA-p21 is transcriptionally regulated by the mutant p53/nuclear transcription factor Y subunit alpha (NF-YA) complex. Low lincRNA-p21 expression promoted aggressive progression in HNSCC in vitro and in vivo. Meanwhile, lincRNA-p21 inhibited Janus kinase 2 (JAK2)/signal transducer and activator of transcription 3 (STAT3) signaling by binding to STAT3 and suppressing its transcriptional activation, which is a novel mechanism of lincRNA-p21. Our findings provide insight into how the p53/lincRNA-p21/STAT3 axis contributes to HNSCC development and indicate that lincRNA-p21 may serve as a novel therapeutic target for HNSCC.

## Methods

### RNAscope, fluorescence in situ hybridization and immunohistochemistry assay

We obtained 70 HNSCC tissues and 9 normal oral mucosal tissues from patients who had undergone surgery between 2007 and 2008 and who were diagnosed by pathological examination. No local or systemic treatment was conducted in these patients before surgery. The tissues were embedded into a tissue microarray. Fresh tumor specimens were collected at surgery and stored at − 80 °C until use. This study was approved by the Ethics Committee of the Ninth People’s Hospital, Shanghai Jiao Tong University School of Medicine.

The RNAscope probe targeting lincRNA-p21 was designed and synthesized by Advanced Cell Diagnostics company, and detection of lincRNA-p21 expression was performed on an HNSCC tissue array using an RNAscope 2.5 High Definition (HD)-BROWN Assay kit according to the manufacturer’s instructions (Advanced Cell Diagnostics, Newark, CA, USA). The images were acquired with a Pannoramic MIDI Viewer (3D HISTECH Ltd., Budapest, Hungary). The signals were visually scored based on the average number of dots per cell using the following criteria: 0 (no staining or < 1 dot/10 cells), 1 (1–3 dots/cell), 2 (4–9 dots/cell. None or very few dot clusters), 3 (10–15 dots/cell and < 10% of the dots presented in clusters), 4 (> 15 dots/cell and > 10% of the dots presented in clusters).

Fluorescence in situ hybridization (FISH) was strictly performed according to the manufacture’s protocol (Ribobio Company, Guangzhou, China). Briefly, target-specific probe was designed, synthesized and incubated overnight at 4 °C. 18S RNA and U6 were used as control to indicate the cytoplasm and nucleus.

Immunohistochemistry (IHC) was performed as described in our previous study [21], with primary antibodies for p-STAT3 (9145) and Ki67 (9129) (1:200 dilution for IHC, Cell Signaling Technology, Danvers, MA, USA). The staining intensity was assessed as follows: 0 = negative; 1 = weak; 2 = moderate; 3 = strong. The staining score was calculated by multiplying the staining intensity and the percentage of positive cells.

### Cell culture

The HNSCC cell lines HN4, HN6 and HN30 were kindly provided by Professor Mao Li, University of Maryland and were verified by STR genotyping, and HEK293T, Cal27, SCC25, Detroit562, MCF7 and MDA-MB-231 cell lines were purchased from Type Culture Collection of Chinese Academy of Sciences (Shanghai, China). Normal oral primary keratinocytes were cultured from gingival tissues after tooth extraction from healthy patients. An informed consent was signed by the patients. SCC25 cells were maintained in Dulbecco’s modified Eagle’s medium (DMEM)/F12, and the other cell lines were maintained in DMEM (Gibco, Grand Island, NY, USA) supplemented with 10% fetal bovine serum, 1% glutamine, and 1% penicillin-streptomycin. Cells were cultured in a standard humidified atmosphere of 5% CO_2_ at 37 °C.

### Cytoplasmic and nuclear RNA isolation and real-time PCR

Cytoplasmic and nuclear RNA was extracted using an RNA Purification Kit (21,000, Norgen Biotek, Thorold, ON, Canada) according to the manufacturer’s instructions. Real-time PCR (qRT-PCR) was performed using a StepOnePlus Real-time PCR system (Thermo Fisher, Waltham, MA, USA) as previously described [22] and following the manufacturer’s instructions (Takara, Dalian, China). The primer sequences are listed in Additional file 1: Table S1.

### Plasmid construction, siRNA, lentivirus and cell transfection

pcDNA3.1 vector containing the human lincRNA-p21 sequence was synthesized by OBiO Technology (Shanghai, China). Plasmids encoding wild-type p53 or mutant p53s (P151S, R175H, G245C and R282W) and lentivirus, including for lincRNA-p21 and si-lincRNA-p21, were synthesized by Gene Pharma Co., Suzhou, China. The lincRNA-p21 promoter reporter gene was cloned into the vector pGL4.0-basic (OBiO Technology, Shanghai, China). Small interfering RNA (siRNA) was synthesized by Biotend Biotechnology, Shanghai, China. The siRNA#3 sequence of lincRNA-21 was packaged into a pGLV-h1-GFP-puro vector for in vivo experiments. Cells were transfected with siRNAs or plasmids using Lipofectamine™ 3000 (Invitrogen, Carlsbad, CA, USA) according to the manufacturer’s instructions. Treatments were administered 24 h after transfection. The sequences are provided in Additional file 1: Table S1.

### Chromatin immunoprecipitation assays

The chromatin immunoprecipitation (ChIP) assay was strictly performed according to the protocol of a SimpleChIP Enzymatic Chromatin IP kit (CST, Danvers, MA, USA) according to the manufacturer’s instructions as described in our previous study [22, 23]. The primers for the lincRNA-p21 promoter were synthetized by Sangon Biotech (Shanghai, China) and are described in Additional file 1: Table S1.

### Dual-luciferase reporter assay

Luciferase assays were performed as described in our previous study [22]. Briefly, HEK293T cells were cotransfected with each lincRNA-p21 promoter-luciferase construct and pRL-TK promoter Renilla luciferase construct in the vector group, wild-type p53 group and mutant p53 group for 48 h. Whole-cell lysates were extracted, and luciferase activity was determined using a dual luciferase reporter assay system (Beyotime, Shanghai, China) according to the manufacturer’s instructions. A pRL-TK promoter Renilla luciferase construct was used as an internal control.

### Electrophoretic mobility shift assay

Electrophoretic mobility shift assay (EMSA) was performed as described previously [24] and according to the instructions of an EMSA kit (Beyotime, Shanghai, China). HN6, Cal27 and HN30 cells were stimulated with or without Doxorubicin (DOX, 500 nM) for 24 h. Total nuclear cell lysate was incubated with 1 pmol biotin-labeled DNA probes (1000 fmol/μl working fluid concentration) with or without 150 pmol cold competitor probes (50 pmol/μl concentration) consisting of a 16-bp region of the lincRNA-p21 promoter. The biotin-labeled probe sequence was Bio-5′-TTGTCCTTGCCTTTGCTTCCCTAGGGT-3′. The unlabeled cold probe sequence was 5′-CTTGTCCTTGCCTTTGCTTCCCTAGGGTG-3′.

### Western blot and immunoprecipitation

Western blotting was performed as described in our previous study [22]. DOX, MG132 and cryptotanshinone were purchased from Selleck (Houston, TX, USA). IL6 was ordered from PeproTech (Rocky Hill, NJ, USA). The antibodies used in this study were as follows: Rb (181616), p-Rb (184796), E2F1 (179445), Caspase-3 (13847) and cleaved Caspases-3 (32042) antibodies were purchased from Abcam (Cambridge, MA, UK). ERK1/2 (4695), p-ERK1/2 (Thr202/Tyr204) (4370), AKT (4691), p-AKT (Ser473) (4060), Cdc2 (28439), Cyclin B1 (4135), Cyclin D1 (2978), PARP (9532), cleaved PARP (5625), p53 (2524), MMP2 (40994), MMP9 (13667), STAT3 (9139), p-STAT3 (Tyr705) (9145), JAK2 (3230) and p-JAK2 (Tyr1007/1008) (3771) antibodies were from Cell Signaling Technology (Danvers, MA, USA). NF-YA (NBP2–19533) was from NOVUS Biologicals (Littleton, USA). GAPDH antibody (60004–1, Proteintech, Rocky Hill, NJ, USA) were used as an internal control. Immunoreactive bands were scanned and analysed using an Odyssey Infrared Imaging System (LI-COR Biosciences, Lincoln, NE, USA).

Immunoprecipitation was operated by incubation with anti-p53 or anti-STAT3 monoclonal antibody with cell lysate at 4 °C rotation overnight. The reaction mix was further incubated with Protein A/G agarose beads for 4 h. The precipitated proteins were detected by immunoblotting with the corresponding antibodies. Anti-GAPDH antibody was used for non-specific control.

### Immunofluorescence assay

Cells were plated on coverslips, and incubated at 4 °C overnight with antibodies specific for p-STAT3 (1:100), NF-YA (1:500) and p53 (1:1000). Then, cells were incubated with Cy3 conjugate goat anti-rabbit IgG (SA00009–2, 1:100) or FITC conjugate goat anti-mouse IgG (SA00003–1) (Proteintech, Hubei, China) and then stained with 4′, 6-diamidino-2-phenylindole (DAPI). Labeled cells were visualized on an Axio Vert. A1 microscope (Carl Zeiss, Germany).

### Cellular proliferation and invasion assay

HN6 and Cal27 cells were transfected with siRNA or plasmids of lincRNA-p21 for 24 h, and then seeded in the plates. As described in our previous study [25], cell proliferation experiments were performed using the Cell Counting Kit (CCK8; Dojindo, Kumamoto, Japan) assays. The colony-forming assay was performed to monitor the cloning capability of HN6 and Cal27 cells [22]. Cell migration and invasion assays were performed using a Transwell technique with uncoated polycarbonate inserts (Millipore, Darmstadt, Germany) or BioCoat™ inserts (BD Biosciences, Franklin Lake, NJ, USA). Medium without FBS (2 × 10^4^ cells/200 μl) were added into the upper portion of a migration (uncoated insert) or invasion (matrigel-coated insert) chamber, with 500 μl DMEM containing 10% FBS added into the lower chamber. After crystal violet staining, the crossed cells were dissolved in 33% of acetic acid. OD 570 nm absorbance was measured.

### 5-Ethyny-2′-deoxyuridine (EdU) assay

The treated HN6 and Cal27 cells were incubated with 50 μM EdU (RiboBio, Guangzhou, China) 100 μl per well for 2 h. After fixation with paraformaldehyde and washes, the cells were treated with 100 μl of 1 × Apollo reaction for 30 min. Then, the DNA of cells were stained with DAPI for 5 min and visualized under Axio Vert. A1 fluorescence microscope.

### Flow cytometry

Flow cytometry was performed using previously described methods [22, 25]. HN6 and Cal27 cells transfected with si-lincRNA-p21, lincRNA-p21, or scramble were harvested 48 h after transfection. After incubating with reagents from the Annexin V-FITC/propidium iodide (PI) apoptosis kit (BD Biosciences, Franklin Lakes, NJ, USA), cells were analysed using a BD FORTASA flow cytometer (BD Biosciences) and the FlowJo software. For cell cycle analysis, cells were incubated using the PI/RNase staining kit (BD Biosciences). The subsequent steps were performed as described above.

### Terminal deoxynucleotidyl transferase dUTP nick end labelling (TUNEL) assay

As described in our previous study [21], TUNEL (Beyotime, Shanghai, China) was performed following the manufacturer’s instructions. Briefly, tissue slice were incubated with fluorescein-labelled dUTP. The nucleus was stained with DAPI. The apoptotic cells (green) were observed and analysed using Axio Vert. A1 fluorescence microscope.

### RNA immunoprecipitation analysis

RNA Immunoprecipitation (RIP) assay was performed according to the manufacturer’s instructions of Millipore Magna RIP Kit (Merck KGaA, Darmstadt, Germany). HN6 cells were transfected with vector or lincRNA-p21 plasmid for 24 h and harvested. The cell lysates were incubated with RIP buffer containing magnetic beads conjugated with control IgG, STAT3, phosphorylated STAT3 antibodies overnight at 4 °C. The samples were then incubated with proteinase K to isolate immunoprecipitated RNA. The isolated RNA was reverse transcribed to cDNA and then analyzed by qRT-PCR. qRT-PCR was performed with SYBR Green PCR (Takara, Dalian, China) and primers targeting lincRNA-p21 truncations (Additional file 1: Table S1).

### RNA pull-down analysis

RNA pull-down assay was performed according to the instructions provided in a Pierce™ Magnetic RNA-Protein Pull-Down Kit (20,164, Thermo Scientific, Waltham, MA, USA). LincRNA-p21 RNA was transcribed in vitro using a DNA template that included the T7 promoter according to the instructions for a Ribo™ RNAmax-T7 Transcription Kit (RiboBio, Guangzhou, China). Then, lincRNA-p21 was end-labeled with desthiobiotin using a Pierce RNA 3′ End Desthiobiotinylation Kit (20,163, Thermo Scientific). HN6 cells were treated with lincRNA-p21 plasmid for 48 h and then harvested. Biotinylated lincRNA-p21 was captured with streptavidin magnetic beads and then mixed with HN6 cell extract. The protein was eluted from the RNA-protein complex and detected by western blot using STAT3 antibody.

### Animal studies

BALB/c nude mice (nu/nu, aged 4 weeks, and weighing approximately 20 g) were purchased from the Shanghai Laboratory Animal Center (Shanghai, China) and were bred in SPF facilities at Shanghai Ninth People’s Hospital, Shanghai Jiao Tong University School of Medicine. The Laboratory Animal Care and Use Committees of the hospital approved all experimental procedures. The tumor xenograft model was established with Cal27 cells as described in our previous study [26]. The Cal27 cells were (2 × 10^6^) stably transduced with lentivirus. The animals were divided into 4 groups: (a) si-Scramble; (b) si-lincRNA-p21; (c) vector; (d) lincRNA-p21. The tumor sizes and animal weights were monitored once a week. The tumor volumes were calculated using the following formula: (length×width^2^)/2. Mice were sacrificed, and the tumor tissues were excised after 6 weeks.

### Statistical analysis

Statistical analyses were performed using SPSS 13.0 software (SPSS Inc., Chicago, IL, USA). GraphPad Prism version 6 (GraphPad Software, San Diego, CA, USA) was used to plot the data. Student’s t-test and one-way analysis of variance (ANOVA) were performed to assess the significance of differences. *P* < 0.05 was considered statistically significant (* *P* < 0.05, and ** *P* < 0.01). All values are expressed as the means ± standard error.

## Results

### Downregulation of LincRNA-p21 is observed in HNSCC and indicates poor prognosis

**Fig. 1:**
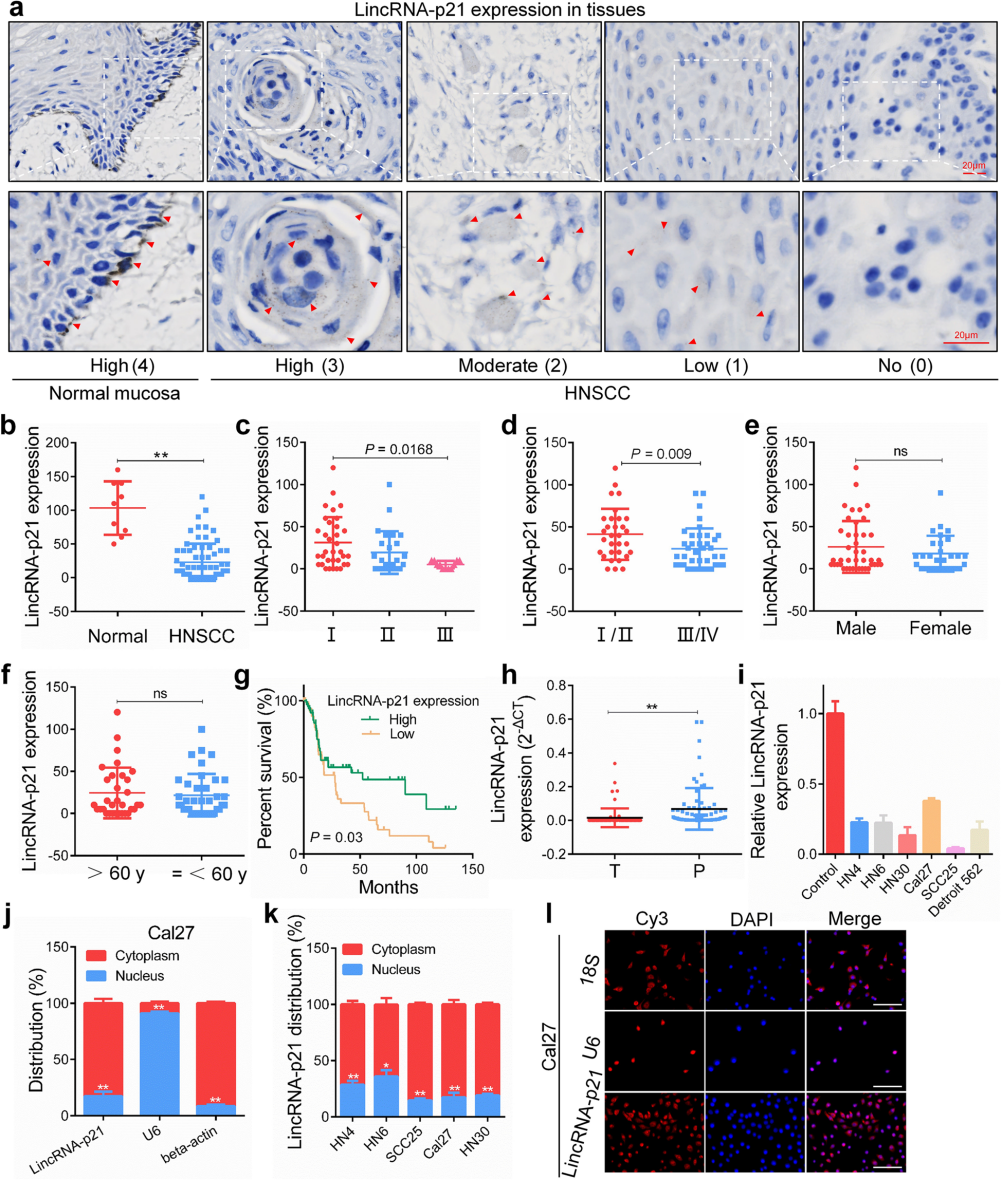
Downregulation of LincRNA-p21 is observed in HNSCC and indicates poor prognosis. **a** The expression of lincRNA-p21 was detected using an RNAscope® in situ assay in normal oral mucosa tissue and OSCC tissues. The red triangles indicate the lincRNA-p21 blots. Scale bars, 20 μm. **b** The lincRNA-p21 staining score in HNSCC patients and normal controls was quantitatively analyzed. **c**-**f** The lincRNA-p21 staining score was analyzed according to pathological differentiation grade, TNM stage, gender and age in HNSCC patients. **g** Survival analysis was performed in HNSCC dataset from TCGA database (http://www.cbioportal.org/). **h** LincRNA-p21 expression was detected in tumor (T) and paracancerous normal tissues (P) from 73 HNSCC patients by qPCR. **i** LincRNA-p21 expression was measured in HNSCC cell lines and normal oral keratinocytes. **j** Localization of lincRNA-p21 was analyzed by PCR in Cal27 cells. U6 RNA and β-actin were used as positive controls for nuclear RNA and cytoplasmic RNA, respectively. **k** The distribution of lincRNA-p21 was analyzed by PCR in HNSCC cell lines. **l** The distribution of lincRNA-p21 was analyzed by FISH in Cal27 cells. 18S RNA and U6 indicated cytoplasm and nucleus, respectively. Scale bars, 100 μm *: *P* < 0.05; **: *P* < 0.01

To investigate the clinical relevance of lincRNA-p21 expression in HNSCC, we first examined 70 HNSCC tissues and 9 normal oral mucosa tissues using an RNAscope assay. RNAscope® is a novel in situ hybridization (ISH) approach for detection of target RNA with a unique probe design strategy that allows simultaneous target-specific signal amplification and background suppression to achieve single-molecule visualization while preserving tissue morphology [27]. We observed that the expression levels of lincRNA-p21 were significantly high in normal mucosa tissues, whereas expression was negligible in most of the HNSCC tissues. Furthermore, lincRNA-p21 was predominantly localized in the cytoplasm and partly distributed in the nuclei (Fig. 1a). The signaling score in HNSCC samples was significantly lower than that in normal oral mucosa samples (22.9 ± 3.3 vs 103.3 ± 13.2, *P* < 0.0001, Fig. 1b). The relationship between lincRNA-p21 and clinical characteristics was analyzed, and tumors with a higher pathological grade had a lower score than those with a lower grade (*P* = 0.0168, Fig. 1c). Moreover, the lincRNA-p21 score was negatively correlated with TNM stage (*P* = 0.009, Fig. 1d). However, there was no association between lincRNA-p21 expression and other factors, including gender (Fig. 1e) and age (Fig. 1f). From TCGA database, we found that HNSCC patients with low lincRNA-p21 expression (median was defined as cutoff value) had worse prognosis (*P* = 0.03, Fig. 1g).

We next conducted qRT-PCR to examine lincRNA-p21 expression in HNSCC tissues and cell lines. LincRNA-p21 was poorly expressed in tumor tissues from 73 patients, but it was highly expressed in matched adjacent non-tumor tissues (Fig. 1h). In addition, the expression of lincRNA-p21 in 6 HNSCC cell lines (HN4, HN6, HN30, Cal27, SCC25 and Detroit562) was lower than in normal oral keratinocytes (Fig. 1i). These findings indicate that low levels of lincRNA-p21 expression may serve as an important regulator in HNSCC progression.

The cellular localization of lncRNAs is intended to enable a range of physiological activities, from chromatin remodeling to translational regulation. Compared with the fractionation indicators β-actin, 18S RNA and U6, lincRNA-p21 was mainly distributed in the cytoplasm in HNSCC cell lines using qPCR and FISH assays (Fig. 1j, k, l).

### NF-YA is necessary for mutant p53 regulation of lincRNA-p21 but not wild-type p53

**Fig. 2:**
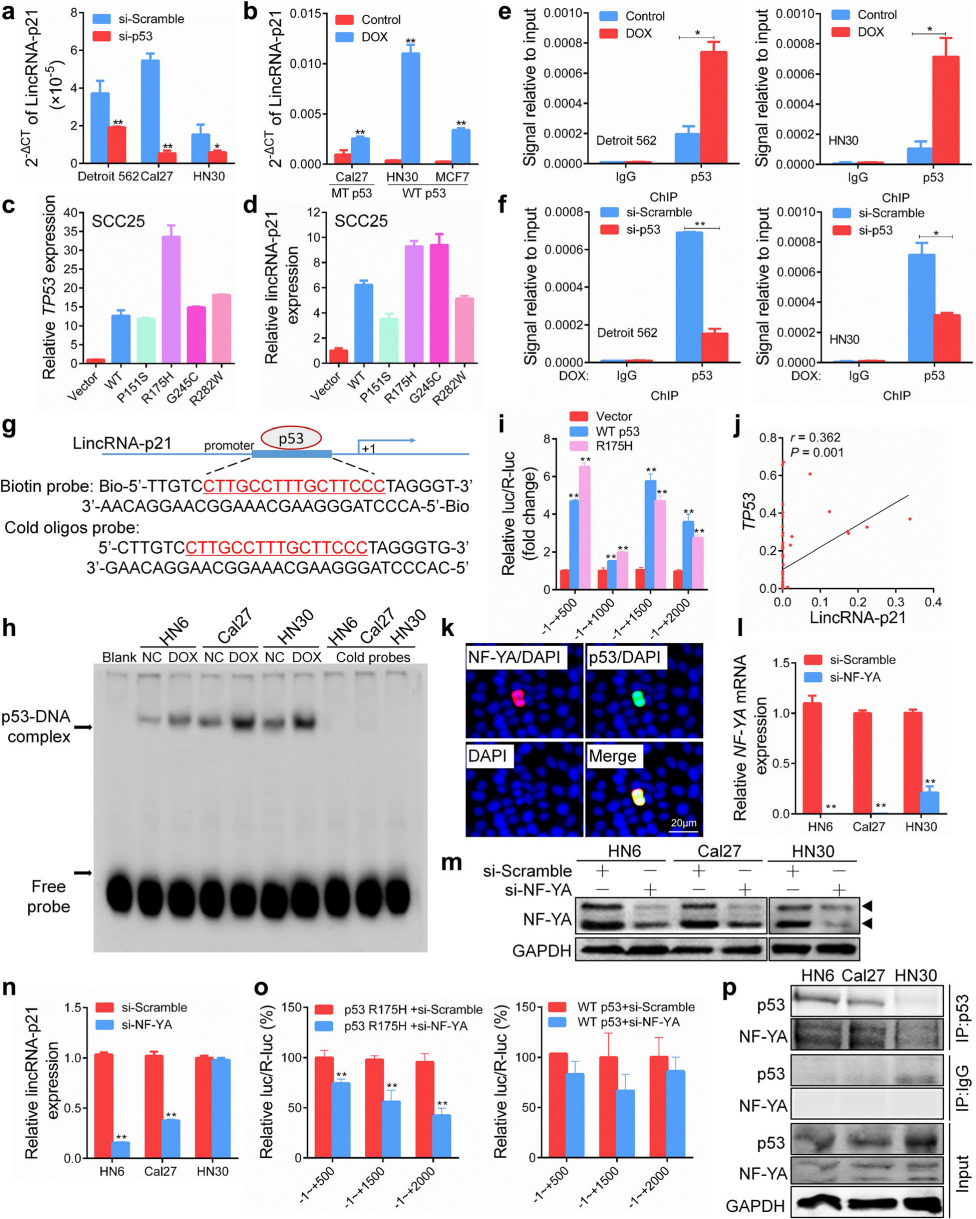
NF-YA is essential for lincRNA-p21 regulation by mutant P53 but not wild-type p53. **a** The expression of lincRNA-p21 was detected by qRT-PCR in cells transfected with si-p53 for 24 h. **b** The expression of lincRNA-p21 was detected by qRT-PCR in cells treated with 500 nM doxorubicin (DOX) for 24 h. **c**, **d** The expression of *TP53* mRNA and lincRNA-p21 were detected by qRT-PCR in SCC25 cells transfected with the wild-type 53 or mutant p53 vector for 24 h. **e**, **f** Detroit562 and HN30 cells were treated with 500 nM DOX or transfected with si-p53 for 48 h. The binding of p53 at the lincRNA-p21 promoter region was detected by a chromatin immunoprecipitation assay. **g** A probe targeting the binding sites of p53 in the lincRNA-p21 promoter was designed according to ChIP assays. **h** HN6, Cal27 and HN30 cells were treated with 500 nM DOX for 48 h. A biotin probe and cold oligo probes were incubated with nuclear extract followed by an EMSA. For the cold competitive EMSA, 150 × nonlabelled probes were used. **i** SCC25 cells were co-transfected with lincRNA-p21 promoter-luciferase truncations and p53 plasmids, and the luciferase activity was determined using a dual luciferase reporter assay after 24 h. **j** The correlation between lincRNA-p21 and *TP53* mRNA was analyzed in 70 HNSCC patients by qPCR. **k** The cellular localization of NF-YA and p53 was visualized by immunofluorescence in HN6 cells. Scale bars, 20 μm. **l**, **m** NF-YA mRNA and protein levels were detected after transfection with siRNA targeting NF-YA. **n** The expression of lincRNA-p21 was detected in HN6, Cal27 and HN30 cells after transfection with si-NF-YA for 24 h. **o** lincRNA-p21 promoter activity was analyzed after co-transfection with p53 R175H or wild-type p53 plasmids and si-NF-YA for 24 h. **p** The binding of p53 to NF-YA in HN6, Cal27 and HN30 cells was determined by coimmunoprecipitation. *: *P* < 0.05; **: *P* < 0.01

Previous studies reported that lincRNA-p21 was a transcription target of wild-type p53 in mouse cells [16]. To determine whether p53 regulates lincRNA-p21 expression in human HNSCC, we firstly analyze the level of lincRNA-p21 after p53 knockdown or overexpression. As shown in Fig. 2a, lincRNA-p21 was significantly inhibited in Detroit 562 and Cal27 cells carrying *TP53* mutations and in HN30 cells with wild-type *TP53* after knockdown of endogenous p53. Furthermore, lincRNA-p21 was strongly upregulated after DNA damage induced by DOX treatment in Cal27 cells and HN30 and MCF7 cells with wild-type *TP53* (Fig. 2b). Furthermore, the expression of lincRNA-p21 was also inhibited after knockdown of p53 in a pair of breast cancer cell lines, MCF7 (wild-type *TP53*) and MDA-MB-231 (mutant *TP53*), which indicated that regulation of lincRNA-p21 by p53 may be a common mechanism in different cancers (Additional file 2: Figure S1). To further confirm this result, we utilized plasmid transfection to exert exogenous expression of wild-type p53 and mutant p53s in p53-null SCC25 cells (Fig. 2c). Compared to the control vector cells, increased lincRNA-p21 expression was observed in SCC25 cells expressing wild-type p53 and mutant p53 (P151S, R175H, G245C and R282W) (Fig. 2d).

We focused on possible transcriptional regulation of lincRNA-p21 by p53 and investigated whether p53 was able to bind the promoter of lincRNA-p21. Using a ChIP assay, we observed that p53 bound to the promoter of lincRNA-p21 under DOX stimulation in Detroit 562 and HN30 cells (Fig. 2e). In addition, silencing p53 attenuated the binding capacity to the lincRNA-p21 promoter in response to DOX (Fig. 2f). An EMSA was used to further determine whether p53 binds to the promoter of lincRNA-p21. A database prediction indicated that the lincRNA-p21 promoter presented a putative binding region for p53 (Fig. 2g). The sequence was confirmed with a ChIP assay. The p53-probe complex bands were significantly increased in DOX-treated cells with the shift probe but not with the cold probe (Fig. 2h). To investigate the underlying mechanism by which wild-type and mutant p53s regulated lincRNA-p21, we measured lincRNA-p21 promoter activity using dual-luciferase assays. Compared with control cells, increased luciferase activities were observed in both wild-type p53- and mutant p53-R175H-transfected 293 T cells (Fig. 2i). Together, these results revealed that lincRNA-p21 was a direct transcriptional target of p53. Furthermore, we observed the positive correlation between lincRNA-p21 and *TP53* mRNA in 70 HNSCC patients (Fig. 2j).

Mutant p53 proteins are known to interact with other cellular transcription factors and thereby enhance the expression of their respective target genes. Since NF-YA has been shown to be an important co-factor of mutant p53 in cancers [28], we investigated the impact of downregulation of NF-YA and its partners. An immunofluorescence assay showed that p53 and NF-YA protein colocalized in the nucleus of HN6 cells under physiological conditions (Fig. 2k). NF-YA mRNA and protein were effectively reduced by siRNA targeting NF-YA in HN6, Cal27 and HN30 cells (Fig. 2l, m). LincRNA-p21 was significantly downregulated after knockdown of NF-YA in HN6 and Cal27 cells carrying *TP53* mutations but not in HN30 with wild-type *TP53* (Fig. 2n). NF-YA knockdown attenuated p53-R175H-induced lincRNA-p21 promoter luciferase activity. However, in wild-type p53 cells, NF-YA knockdown did not affect p53-induced lincRNA-p21 promoter luciferase activity (Fig. 2o). An immunoprecipitation assay revealed that p53 bound to NF-YA protein in HN6 and Cal27 cells but not in HN30 cells (Fig. 2p). These data suggest that NF-YA is necessary for mutant p53 regulation of lincRNA-p21 but not wild-type p53.

### LincRNA-p21 suppresses HNSCC tumor growth in vitro and in vivo

**Fig. 3:**
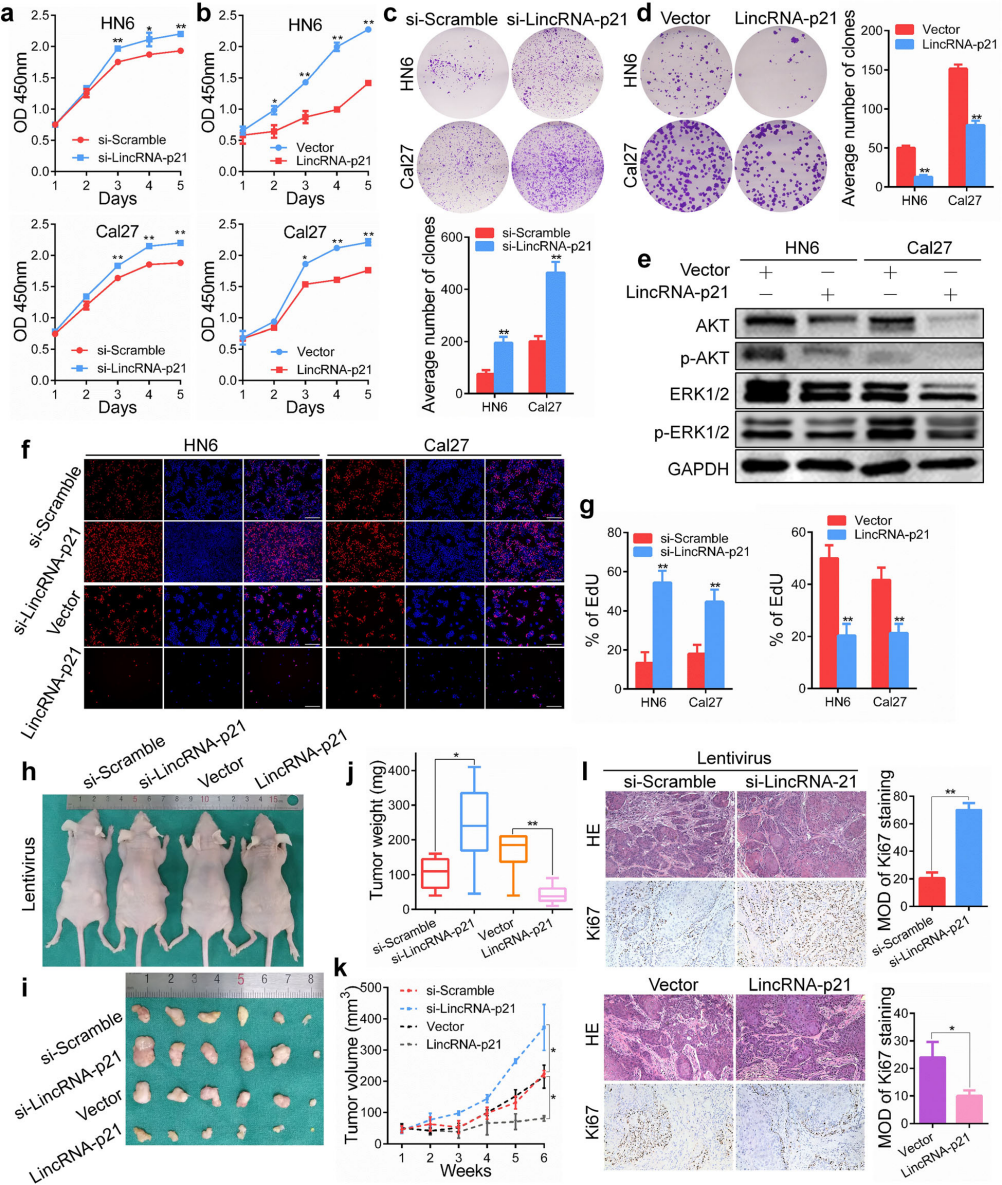
LincRNA-p21 suppresses HNSCC tumor growth in vitro and in vivo*.* **a** Cell viability of HN6 and Cal27 cells after transfection with si-lincRNA-p21 or scrambled was determined using CCK8 assays. **b** Cell viability of lincRNA-p21- or vector-transfected HN6 and Cal27 cells was determined using CCK8 assays. **c** A colony formation assay was used to determine the colony formation ability of si-lincRNA-p21- or scrambled-transfected HN6 and Cal27 cells. **d** The colony formation assay was applied in HN6 and Cal27 cells after transfection with lincRNA-p21 or vector plasmids. **e** AKT and ERK1/2 signaling was analyzed via western blotting after transfection for 48 h. **f**, **g** EdU assays were performed after transfection of HN6 and Cal27 cells for 48 h. Scale bars, 100 μm. **h**, **i** Representative images of xenograft tumors derived from si-lentivirus lincRNA-p21, lentivirus lincRNA-p21 or scrambled cells were shown. **j** The tumor weight after removal from mice in the knockdown, ectopic expressed and scrambled control groups was measured. **k** The tumor growth curves after cell injection in the si-lentivirus lincRNA-p21, lentivirus lincRNA-p21 or scrambled group were analyzed. **l** HE and Ki67 staining were detected by immunohistochemistry in the xenograft tumor tissues. Original magnification: × 200. *: *P* < 0.05; **: *P* < 0.01

To determine the functional role of lincRNA-p21 in HNSCC biological behavior, we investigated the effects of lincRNA-p21 down-regulation or upregulation on cell proliferation. We silenced lincRNA-p21 expression in HN6 and Cal27 cells which had relative high expression of lincRNA-p21 among the HNSCC cell lines with small interfering RNA (siRNA), which reduced the mRNA expression levels of lincRNA-p21 (Additional file 2: Figure S2a). Knockdown of lincRNA-p21 significantly increased cell proliferation and colony formation compared with the scrambled control in both HN6 and Cal27 cell lines (Fig. 3a, c). Similar results were achieved with lincRNA-p21 antisense oligonucleotide (ASO) treatment (Additional file 2: Figure S3a, b). Cells were transfected with plasmid for lincRNA-p21 overexpression (Additional file 2: Figure S2b). Concordantly, cell proliferation and colony formation were inhibited by overexpression of lincRNA-p21 (Fig. 3b, d). Furthermore, ectopic expression of lincRNA-p21 also inhibited proliferation and colony formation capacity in HN30 cells harboring wild-type p53 (Additional file 2: Figure S4a, b). We next investigated potential mechanisms by which lincRNA-p21 overexpression could inhibit OSCC proliferation. We analyzed the expression of a spectrum of key proliferation and stress-related proteins, including AKT, p-AKT, ERK1/2 and p-ERK1/2, in HN6 and Cal27 cells via western blot. We found that overexpression of lincRNA-p21 reduced AKT and ERK1/2 production and phosphorylation (Fig. 3e). Notably, these molecules were altered in an opposite manner when lincRNA-p21 was depleted using siRNA (Additional file 2: Figure S5). In addition, an EdU assay was performed to determine cell DNA replication activity and cell proliferation. A significant inhibition of cell proliferating activity was observed in ectopic expression of lincRNA-p21, and vice versa (Fig. 3f, g).

To verify whether lincRNA-p21 can affect HNSCC progression in vivo, subcutaneous tumor growth of Cal27 cells was monitored. Tumor growth accelerated in the lincRNA-p21 knockdown group compared with the scrambled control; however, growth was impeded in the lincRNA-p21 overexpression group (Fig. 3h, i). The transfection efficiency was confirmed in fresh xenograft tissue using qPCR (Additional file 2: Figure S6).The tumor weight and volume were greatly increased in lincRNA-p21-depleted mice but were reduced in the lincRNA-p21-overexpressed mice compared with control mice (Fig. 3j, k). In addition, hematoxylin and eosin (HE) staining and Ki67 staining were performed to verify the results. Compared to their respective control groups, the expression of Ki67 protein was higher in the knockdown group and lower in the overexpression group (Fig. 3l). These findings indicate that lincRNA-p21 acts as a tumor suppressor lncRNA in HNSCC.

### Overexpression of lincRNA-p21 inhibits G1/S transition and induces apoptosis in HNSCC cells

**Fig. 4:**
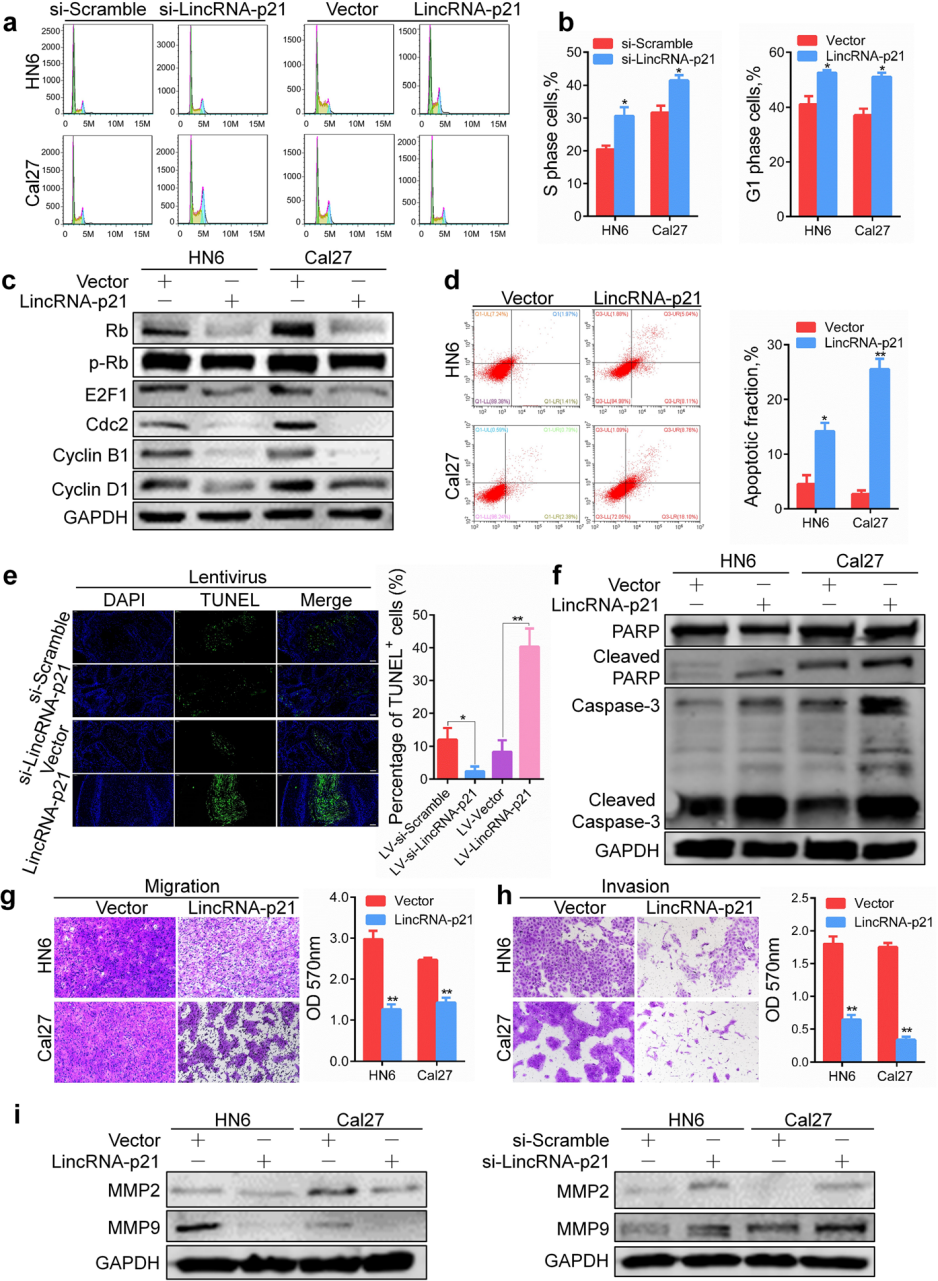
LincRNA-p21 regulates cell cycle and induces apoptosis in HNSCC cells. **a**, **b** Cell cycle distribution was determined by PI staining after transfection of HN6 and Cal27 cells with siRNA or lincRNA-p21 plasmid for 48 h. **c** Cell cycle regulation-related proteins were detected in HN6 and Cal27 cells after transfection for 48 h. **d** The proportion of apoptotic cells was detected after lincRNA-p21 plasmid transfection for 48 h using PI and Annexin V staining. **e** The proportion of apoptotic cells was detected by TUNEL assays in the xenograft tumor tissues. Scale bars, 100 μm. **f** PARP, Caspase-3 and its active forms were detected in HN6 and Cal27 cells after transfection for 48 h. **g**, **h** Migration and invasion assays were performed with transfected cells using Transwell inserts. **i** MMP2 and MMP9 expression was detected in HN6 and Cal27 cells after transfection for 48 h. *: *P* < 0.05; **: *P* < 0.01

We investigated whether linRNA-p21 is involved in regulation of cell cycle and apoptosis by performing flow cytometry. The results showed that knockdown of lincRNA-p21 increased the percentage of S phase cells, while overexpression of lincRNA-p21 promoted G1 arrest in HN6 and Cal27 cells (Fig. 4a, b). Furthermore, overexpression of lincRNA-p21 decreased the expression levels of cell cycle regulating proteins (Rb, p-Rb, E2F1, Cdc2, Cyclin B1 and Cyclin D1) (Fig. 4c). Knockdown of lincRNA-p21 showed the opposite results (Additional file 2: Figure S7a). These results indicate that overexpression of lincRNA-p21 inhibits G1/S transition to impede cell cycle progression.

Flow cytometry analysis revealed that overexpression of lincRNA-p21 significantly induced apoptosis in HNSCC cells (Fig. 4d). The percentage of apoptotic cells was increased in xenograft tissue from the lincNRA-p21-overexpressed group compared with the control vector group, while knockdown of lincRNA-p21 reduced apoptosis (Fig. 4e). Consistent with the flow cytometry results, western blot showed that the expression of the apoptosis markers cleaved-PARP and cleaved-caspase 3 were significantly upregulated in lincRNA-p21 overexpression cells (Fig. 4f). Knockdown of lincRNA-p21 showed the opposite results (Additional file 2: Figure S7b). Moreover, transwell assays were performed to determine whether lincRNA-p21 could affect the aggressiveness migration of HNSCC. Ectopic expression of lincRNA-p21 decreased the migration and invasion capacity of HN6 and Cal27 cells, and vice versa (Fig. 4g, h, Additional file 2: Figure S8a, b). Correspondingly, matrix metalloproteinase 2 (MMP2) and MMP9 protein were reduced after lincRNA-p21 overexpression but increased after the silencing of lincRNA-p21 (Fig. 4i). These findings demonstrate that ectopic expression of lincRNA-p21 inhibits aggressive manners in HNSCC cells.

### Knockdown NF-YA expression reverses tumor suppressor activation of lincRNA-p21 in mutant p53 cells, not wild-type p53 cells

**Fig. 5:**
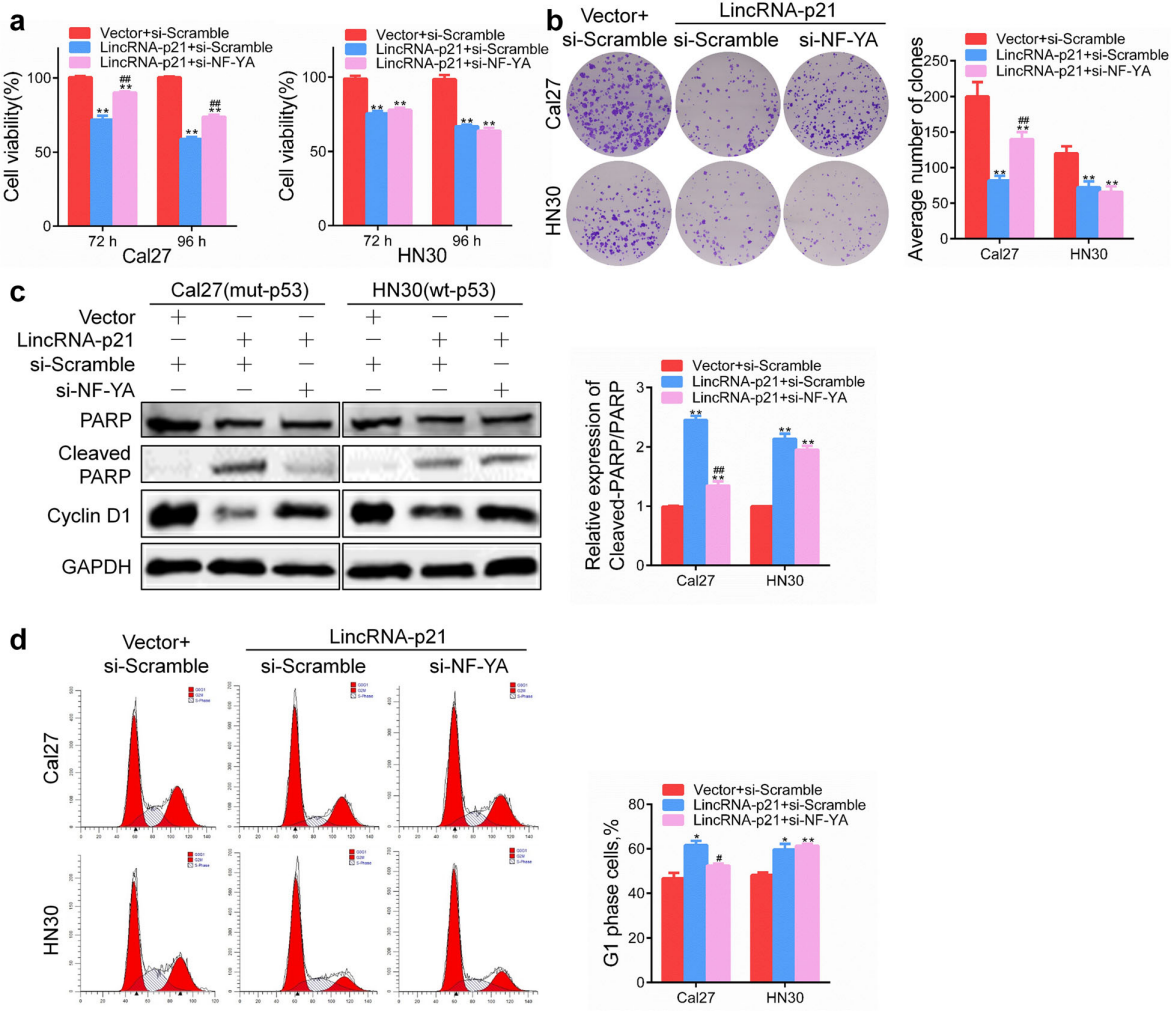
Knockdown NF-YA expression reverses tumor suppressor activation of lincRNA-p21 in mutant p53 cells, not wild-type p53. **a** Cell viability was detected using CCK8 in Cal27 (mut-p53) and HN30 (wt-p53) cells after co-transfection with lincRNA-p21 and si-NF-YA for 24 h. **b** Colony formation was conducted in Cal27 and HN30 cells after co-transfection with lincRNA-p21 and si-NF-YA. **c** Cleaved PARP and Cyclin D1 was detected using western blot after co-transfection with lincRNA-p21 and si-NF-YA for 48 h in Cal27 and HN30 cells. **d** Cell cycle distribution was determined by PI staining after co-transfection for 48 h. *: *P* < 0.05 and **: *P* < 0.01 indicated the significance compared to the vector control. ^#^: *P* < 0.05 and ^##^: *P* < 0.01 indicated the significance between lincRNA-p21 overexpression groups

As mentioned, NF-YA is an important regulator for mutant p53-relugated lincRNA-p21 expression. To further elucidate the role of NF-YA/p53/lincRNA-p21 signaling in HNSCC cells, Cal27 (mut-p53) and HN30 (wt-p53) cells after co-transfection with lincRNA-p21 plasmids and si-NF-YA were analyzed to observe that knockdown NF-YA expression could reverse the inhibition of proliferation capacity of lincRNA-p21 overexpression in Cal27 cells, not HN30 cells (Fig. 5a, b). Cleaved PARP expression was also reduced in lincRNA-p21 and si-NF-YA co-transfection cells in Cal27, while has no effect in HN30 cells (Fig. 5c). Cyclin D1 is the key regulator of G1/S transition to promote cell cycle progression. Inhibition of Cyclin D1 mediated by ectopic expression of lincRNA-p21 was rescued by knockdown NF-YA only in Cal27 cells, while not obvious in HN30 cells (Fig. 5c). Furthermore, overexpression of lincRNA-p21 increased the percentage of G1 phase cells, while si-NF-YA decreased it in Cal27, not in HN30 cells (Fig. 5d). These results demonstrate that si-NF-YA can reverse tumor suppressor activation of lincRNA-p21 in mutant p53 cells, not wild-type p53 cells.

### LincRNA-p21 inhibits JAK2/STAT3 phosphorylation

**Fig. 6:**
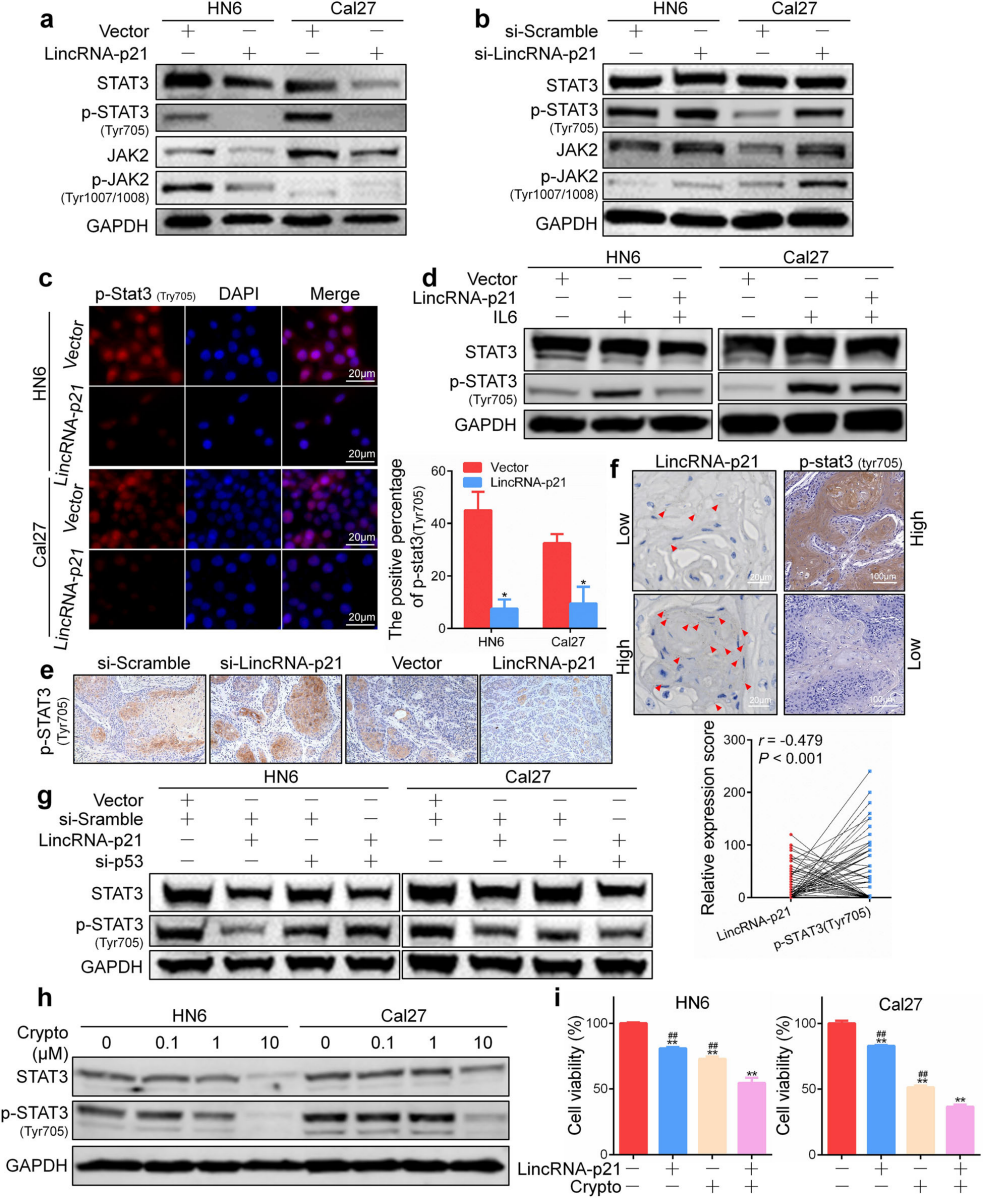
LincRNA-p21 inhibits JAK2/STAT3 activation in HNSCC cells. **a**, **b** The protein levels of STAT3, p-STAT3, JAK2 and p-JAK2 were detected in HN6 and Cal27 cells transfected with lincRNA-p21 plasmid or si-lincRNA-p21 for 48 h. **c** The expression of p-STAT3 in HN6 and Cal27 cells transfected with lincRNA-p21 plasmid for 48 h was visualized by immunofluorescence. **d** The expression of STAT3 and p-STAT3 protein was detected after transfection of HN6 and Cal27 cells for 24 h followed by stimulation with 50 ng/ml IL6 for 30 min. **e** p-STAT3 was detected by immunohistochemistry in the xenograft tumor tissues. Original magnification: × 200. **f** The correlation between lincRNA-p21 and p-STAT3 expression in HNSCC tissues was detected and analyzed using RNAscope and immunohistochemistry, respectively. Scale bars, 20 μm and 100 μm. **g** The expression of STAT3 and p-STAT3 protein were detected after co-transfection with lincRNA-p21 and si-p53 for 48 h. **h** After treatment with STAT3 inhibitor cryptotanshinone (crypto) for 24 h, STAT3 and p-STAT3 expression were detected by western blot in HN6 and Cal27 cells. **i** Cell viability was detected using CCK8 after transfection with lincRNA-p21 and incubation with 1 μM cryptotanshinone for 72 h. **: *P* < 0.01 indicated the significance compared to the control and ^##^: *P* < 0.01 indicated the significance compared to the combination group

Abnormal activation of the JAK2/STAT3 pathway plays critical roles in tumor cell proliferation, invasion, survival, angiogenesis and immunosuppression [29]. Overexpression of lincRNA-p21 significantly decreased the STAT3, p-STAT3, JAK2 and p-JAK2 expression in both HN6 and Cal27 cells. In particular, overexpression of lincRNA-p21 almost completely inhibited STAT3 phosphorylation at tyrosine 705 (Fig. 6a). To detect whether the decrease in STAT3 is dependent on ubiquitination, MG132, a proteasome inhibitor, was applied and no ubiquitin accumulation was found in HNSCC cells (Additional file 2: Figure S9). Moreover, silencing of lincRNA-p21 promoted phosphorylation of STAT3 and JAK2 (Fig. 6b). Similarly, overexpression of lincRNA-p21 reduced p-STAT3 expression in HN6 and Cal27 cells according to an immunofluorescence assay (Fig. 6c). IL-6 is produced by numerous cell types located within the tumor microenvironment, and it acts directly on tumor cells to mediate STAT3 tyrosine phosphorylation and induce the expression of STAT3 target genes [30]. To further determine whether lincRNA-p21 could protect STAT3 from IL-6-induced phosphorylation, cells were transfected with lincRNA-p21 plasmids and then stimulated with IL6. We found that ectopic expression of lincRNA-p21 efficiently abolished the IL-6-induced STAT3 phosphorylation in HN6 cells. Consistently, the phosphorylation of STAT3 induced by IL6 stimulation was partially reversed by overexpression of lincRNA-p21 in Cal27 cells (Fig. 6d). A subsequent IHC analysis verified the lower expression levels of p-STAT3 in lincRNA-p21-overexpressed xenografts compared to control xenografts (Fig. 6e; Additional file 2: Figure S10), indicating that the lincRNA-p21-STAT3 axis plays an important role in carcinogenesis. Furthermore, we analyzed the expression of lincRNA-p21 and p-STAT3 using HNSCC tissue arrays. As shown in Fig. 6f, lincRNA-p21 level negatively correlated with that of p-STAT3 in HNSCC tissues (*P*<0.001). According to our abovementioned results, p53 can transcriptionally activate lincRNA-p21 expression. Thus, we wondered whether p53 can regulate the expression of STAT3 through lincRNA-p21. As shown in Fig. 6g, the increase in STAT3 mediated by p53 knockdown can be attenuated by overexpression of lincRNA-p21 in HN6 and Cal27 cells. To test whether lincRNA-p21 inhibited HNSCC cell growth through JAK2/STAT3 signaling, a STAT3 inhibitor cryptotanshinone was applied. It can markedly inhibited the expression of STAT3 and p-STAT3 in HN6 and Cal27 cells (Fig. 6h; Additional file 2: Figure S11). Cell viability was significantly inhibited in lincRNA-p21 and cryptotanshinone combination group compared to each alone (Fig. 6i).

### LincRNA-p21 binds to STAT3 to inhibit its phosphorylation

**Fig. 7:**
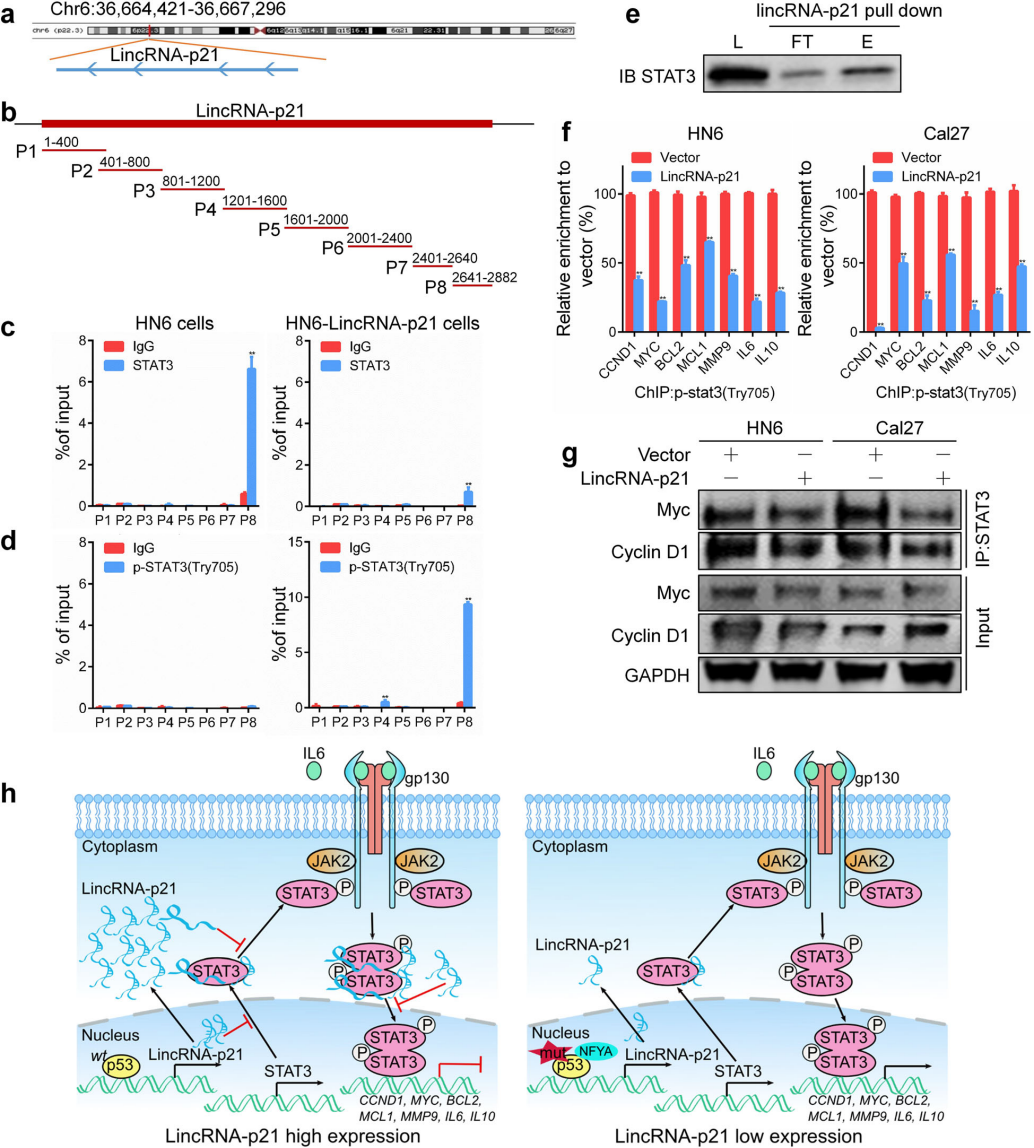
LincRNA-p21 directly binds to STAT3, impeding its cancer-promoting activity in HNSCC. **a** The chromosomal location of human lincRNA-p21 was shown. **b** Eight consecutive primers were designed to amplify the full-length human lincRNA-p21 sequence. **c** HN6 cells were transfected with lincRNA-p21 plasmid and vector for 48 h. RNA immunoprecipitation was used to detect the binding of STAT3 and lincRNA-p21. **d** HN6 cells were transfected with lincRNA-p21 plasmid and vector for 48 h. RNA immunoprecipitation was used to detect the binding of p-STAT3 and lincRNA-p21. **e** Biotinylated lincRNA-p21 was incubated with cell lysates, and the eluent and flow-through were detected via western blotting using anti-STAT3 antibody. L: lysate load; FT: flow-through; E: eluate. **f** The binding of p-STAT3 to the CCND1, MYC, BCL2, MCL1, MMP9, IL6 and IL10 promoters was detected by a chromatin immunoprecipitation assay in HN6 and Cal27 cells after lincRNA-p21 transfection for 48 h. **g** The binding of STAT3 and Myc or cyclin D1 was determined by an immunoprecipitation assay in HN6 and Cal27 cells after transfection with lincRNA-p21 plasmid for 48 h. **h** Schematic diagram show that lincRNA-p21 plays a critical role in the p53/lincRNA-p21/JAK2/STAT3 axis promoting malignant disease progression in HNSCC. *: *P* < 0.05; **: *P* < 0.01

Using the UCSC database, we identified the location and sequence of human lincRNA-p21 (Fig. 7a). Eight truncated primers were used to amplify the full-length lincRNA-p21 sequence (Fig. 7b). Based on an RIP assay, STAT3 directly bound to lincRNA-p21 at the P8 fragment in HN6 cells. Both STAT3 and p-STAT3 exhibited significant binding to lincRNA-p21 in HN6-lincRNA-p21 cells (Fig. 7c, d). RNA pull-down assays using biotinylated lincRNA-p21 were conducted to confirm the specific interaction between STAT3 and lincRNA-p21 in HN6 lysates (Fig. 7e). We next investigated whether lincRNA-p21 could regulate the well-known target genes of STAT3. As shown in Fig. 7f, overexpression of lincRNA-p21 significantly attenuated the enrichment of the CCND1, MYC, BCL2, MCL1, MMP9, IL6 and IL10 promoters according to a ChIP assay. In addition, an IP assay showed that ectopic expression of lincRNA-p21 inhibited the interaction between STAT3 and Myc or Cyclin D1 protein in HN6 and Cal27 cells (Fig. 7g). These data indicate that lincRNA-p21 directly binds to STAT3 to impede its protumorigenic signals in HNSCC. In short, high expression of lincRNA-p21 increases its binding to STAT3, inhibiting STAT3 transcriptional activity and suppressing tumor progression and vice versa (Fig. 7h).

## Discussion

In this study, we first investigated the difference between wild-type and mutant p53 in regulating lincRNA-p21 expression in HNSCC. Moreover, high lincRNA-p21 expression exerted tumor suppressor activity by inhibiting JAK2/STAT3 signaling activation, which suggest that lincRNA-p21 was deeply involved in aggressive progression in HNSCC.

LincRNA-p21 is a relatively novel lncRNA that plays a significant role in the initiation and development of multiple cancers. Previous studies have reported that lincRNA-p21 was significantly downregulated in hepatocellular carcinoma [31], colorectal cancer [32], gastric cancer [33], prostate cancer [34], non-small cell lung cancer [35], diffuse large B cell lymphoma [36], and chronic lymphocytic leukemia [37]. In addition, the exosomal lincRNA-p21 levels in urine were significantly higher in prostate cancer than in benign prostatic hyperplasia [38]. In our study, we first demonstrated that lincRNA-p21 was reduced in HNSCC tissues and cell lines compared with normal mucosa. Although we did not obtain the survival data of the patients in our study, we found that HNSCC patients with low lincRNA-p21 expression had worse prognosis using TCGA database. The relationship between the expression level of lincRNA-p21 and prognosis is heterogeneous in different types of tumors. High expression of lincRNA-p21 was linked to favorable survival in diffuse large B lymphoma patients and hepatocellular carcinoma patients, whereas lincRNA-p21 acted as a tumor suppressor, inhibiting cell growth, arresting cycle progression and facilitating apoptosis [36, 39]. However, in lung cancer, patients with high lincRNA-p21 levels had a worse prognosis than those with low levels [35]. We showed that lincRNA-p21 inhibited HNSCC cell growth in vitro and in vivo. Furthermore, lincRNA-p21 induced G1 phase arrest and cell apoptosis. Thus, lincRNA-p21 is considered as a tumor suppressor in most of tumors reported thus far, but how lincRNA-p21 promotes tumor progression in HNSCC needs further study.

p53 is a sequence-specific DNA-binding protein that interacts with promoter regions of target genes and regulates transcription. Accumulating evidences indicate that p53 not only transcribes messenger RNAs but also noncoding RNAs, including microRNAs and long noncoding RNAs. Liu et al. [40] reported that lncRNA loc285194 was induced by p53 and inhibited tumor cell growth. Under glucose starvation conditions, p53 directly upregulated lncRNA TRINGS and protected cancer cells from necrosis [41]. Consistent with the function of p53-mediated transcription, *TP53* missense mutation mainly occurs in its DNA-binding domain [42]. Mutations that perturb p53 function or disrupt the upstream or downstream regulatory network of p53 have been found in more than half of all cancer cases [43]. Even in those cancers that retain wild-type p53, the function of p53 is often inactivated due to alterations in its regulators and/or mediators [44]. However, the molecular mechanisms whereby mutant p53 regulates gene expression are still unclear. Previous studies have reported that four “hot spot” p53 mutation sites (i.e., P151S, R175H, G245C and R282W) promoted invasive growth of HNSCC cells [19]. We chose these four mutant p53 types for further study. In the current study, we showed that both wild-type p53 and mutant p53 promoted the expression of lincRNA-p21 in HNSCC cells, especially after stimulation with DOX. The interaction between p53 and lincRNA-p21 was affirmed by p53 binding to the predicted site of the promoter region of lincRNA-p21 and by p53 causing significant induction of lincRNA-p21 promoter activity, as determined by a luciferase reporter assay. An intriguing question is why both wild-type p53 and mutant p53 promote lincRNA-p21 expression. This may be attributed to the lower expression of lincRNA-p21 in HNSCC patients harboring mutant p53 than in normal tissues with wild-type p53. Mutant p53 loses the ability to bind DNA sequences specific to wild-type p53, but it is widely accepted that mutant p53 is still able to influence gene expression through other transcriptional co-factors, such as p63, p73, nuclear transcription factor Y (NF-Y) and vitamin D receptor [45]. We propose that certain molecules must be involved in regulating lincRNA-p21 expression mediated by mutant p53. Our results demonstrated that mutant p53 transcriptionally regulates lincRNA-p21 depending on NF-YA. Knockdown of NF-YA inhibited the binding affinity between p53-R175H and the lincRNA-p21 promoter but did not affect wild-type p53 function. Knockdown NF-YA expression reversed tumor suppressor activation of lincRNA-p21 in mutant p53 cells, not wild-type p53 cells. These results illustrated that NF-YA plays a critical role in regulation of lincRNA-p21 by mutant p53 but not wild-type p53 in HNSCC.

LncRNAs impact cellular functions through various mechanisms, such as interaction with chromatin remodeling proteins, genomic DNA, mRNA or proteins in the nucleus or cytoplasm [46]. The basic structural and interactive capabilities of lncRNAs with other cellular biomolecules can help distinguish and specifically reveal their central roles in tumorigenesis. Previous investigations have described diverse cellular functions of lincRNA-p21. Hypoxia-induced lincRNA-p21 attenuated VHL-mediated HIF-1α ubiquitination and promoted glycolysis [4]. LincRNA-p21 acted in concert with hnRNP-K as a coactivator to promote p53-mediated expression of p21 [3]. In the absence of HuR, lincRNA-p21 associated with CTNNB1 and JUNB mRNA, and repressed their translation through a mechanism that involves reduced polysome size [47]. How lincRNA-p21 promotes malignant progression in HNSCC is still obscure. The interactions of lncRNAs with RNA, DNA and protein are involved in various levels of regulation, including transcriptional repression by binding to polycomb repressive complex 2 and serving as a sponge to titrate miRNAs, thus participating in posttranscriptional processing. All the classic molecular mechanisms of lncRNAs, such as guiding, scaffolding and decoying, are ultimately executed through interactions with proteins. The JAK-STAT pathway was originally discovered in the context of interleukin-6 (IL-6)-mediated downstream signaling [48]. In fact, the hyperactivation of STAT3 signaling occurs in the majority of human cancers and is correlated with a poor prognosis [29]. Our data showed that p-STAT3 expression was higher in HNSCC tissues and was negatively correlated with lincRNA-p21. Ectopic expression of lincRNA-p21 repressed the IL6-induced STAT3 phosphorylation. LincRNA-p21 was mainly distributed in the cytoplasm of HNSCC cells. Moreover, an RIP assay showed that STAT3 but not p-STAT3 interacted with endogenous lincRNA-p21 under normal conditions; however, p-STAT3 mainly combined with lincRNA-p21 in lincRNA-p21-overexpressed cells. Under normal physiological conditions, STAT3 expression was high in HN6 cells, whereas p-STAT3 was relatively low (Fig. 6a). So lincRAN-21 mainly binds to STAT3 in HN6 cells, which may attribute to competitive advantage of expression. While ectopic expression of lincRNA-p21 was more sensitive to p-STAT3 to bind and inhibit its expression. This is why overexpression of lincRNA-p21 can significantly inhibit p-STAT3 expression, while slightly in STAT3 in HN6 cells. This binding shift can explain the inhibition of phosphorylation of STAT3 after lincRNA-p21 overexpression in HNSCC. The interaction of biotinylated lincRNA-p21 with STAT3 was further confirmed by an RNA pull-down assay. Ectopic expression of lincRNA-p21 also transcriptionally impeded STAT3-regulated gene expression. These data demonstrated that lincRNA-p21 interacts with STAT3 and blocks STAT3 phosphorylation, thereafter repressing the STAT3-regulated downstream genes in HNSCC. Activation of JAK2/STAT3 signaling may serve as the main mechanism that drives tumor progression of low expression of lincRNA-p21 in HNSCC.

## Conclusions

In conclusion, our findings showed that mutant p53 bound to the promoter of lincRNA-p21 and activated the transcription of lincRNA-p21 depending on the co-factor NF-YA. We further demonstrated that lincRNA-p21 acts as a tumor suppressor via direct binding to STAT3 and inhibition of its transcriptional activation. Overall, the p53/NF-YA/lincRNA-p21/STAT3 axis is a novel mechanism that drives malignant progression and thus may be an attractive therapeutic target in HNSCC.

## Additional files

Additional file 1: **Table S1.** The primers and sequences for qPCR, siRNA, ChIP and RIP assays. (XLSX 12 kb)

Additional file 2 **Figure S1.** The expression of lincRNA-p21 was detected by qPCR in breast cancer cells transfected with si-p53 for 24 h. **Figure S2.** (a) Transfection efficiency was detected after siRNA using qPCR in HN6 and Cal27 cells. (b)Transfection efficiency was detected after expression plasmid transfection using qPCR in HN6, HN30 and Cal27 cells. **Figure S3.** Cell viability (a) and colony formation ability (b) of HN6 and Cal27 cells after transfection with ASO-lincRNA-p21 or scrambled were determined using the CCK8 and colony formation assay. **Figure S4.** Cell viability (a) and colony formation ability (b) of HN30 cells after transfection with lincRNA-p21 or vector were detected using the CCK8 and colony formation assay. **Figure S5.** AKT and ERK1/2 signaling were analysed using western blot after si-lincRNA-p21 transfection for 48 h. **Figure S6.** The transfection efficiency was confirmed in fresh xenograft tissue using qPCR. **Figure S7.** LincRNA-p21 regulates cell cycle and apoptosis related protein in HNSCC cells. (a) The cell cycle regulation related proteins were detected in HN6 and Cal27 cells after si-lincRNA-p21 for 48 h. (b) PARP, Caspase-3 and its active forms were detected in HN6 and Cal27 cells after si-lincRNA-p21 for 48 h. **Figure S8.** Migration (a) and invasion (b) assays were performed with si-lincRNA-p21 or scrambled transfected HN6 and Cal27 cells using Transwell inserts. **Figure S9.** LincRNA-p21 reducing STAT3 expression is independent on ubiquitination degradation. Expression of STAT3 and Ubiquitin protein was detected after transfection for 48 h and then stimulation with 0.5 μM MG132 for 24 h in HN6 and Cal27 cells. **Figure S10.** The staining score of p-STAT3 in in the xenograft tumour tissues. **Figure S11.** IC50 was calculated using cryptotanshinone (a STAT3 inhibitor) at indicated concentrations for 72 h in HN6 and Cal27 cells. (DOCX 1296 kb)

## Abbreviations

ChIP: Chromatin Immunoprecipitation
DAPI: 4′, 6-diamidino-2-phenylindole
DOX: Doxorubicin
EMSA: Electrophoretic mobility shift assay
FISH: Fluorescence in situ hybridization
H&E: Hematoxylin and Eeosin
HNSCC: Head and neck squamous cell carcinoma
JAK2: Janus kinase 2
LincRNA-p21: long intergenic non-coding RNA p21
LncRNA: Long non-coding RNA
NF-YA: Nuclear transcription factor Y subunit alpha
qRT-PCR: Quantitative real-time polymerase chain reaction
RIP: RNA Binding Protein Immunoprecipitation
STAT3: signal transducer and activator of transcription 3
TUNEL: Terminal deoxynucleotidyl transferase dUTP nick end labelling
