# Supplementary material for: p53-targeted lincRNA-p21 acts as a tumor suppressor by inhibiting JAK2/STAT3 signaling pathways in head and neck squamous cell carcinoma
Source: Mol Cancer. 2019 Mar 11;18:38. doi: 10.1186/s12943-019-0993-3 (PMC6410525; doi:10.1186/s12943-019-0993-3)

**Supplementary Figures and Figure legends**

**Fig. S1** The expression of lincRNA-p21 was detected by qPCR in breast cancer cells transfected with si-p53 for 24 h.

**

**

**Fig. S2**. (a)Transfection efficiency was detected after siRNA using qPCR in HN6 and Cal27 cells. (b)Transfection efficiency was detected after expression plasmid transfection using qPCR in HN6, HN30 and Cal27 cells.


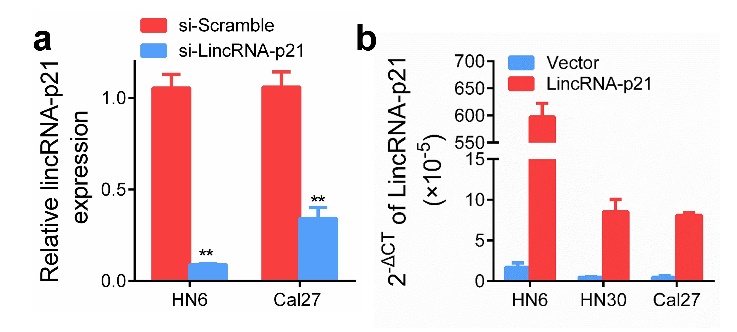


**Fig. S3** Cell viability (a) and colony formation ability (b) of HN6 and Cal27 cells after transfection with ASO-lincRNA-p21 or scrambled were determined using the CCK8 and colony formation assay.


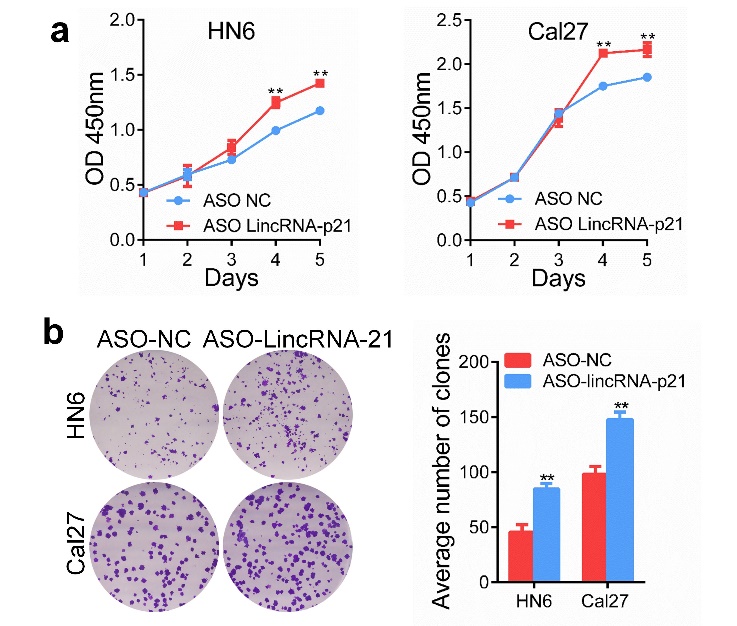


**Fig. S4** Cell viability (a) and colony formation ability (b) of HN30 cells after transfection with lincRNA-p21 or vector were detected using the CCK8 and colony formation assay.


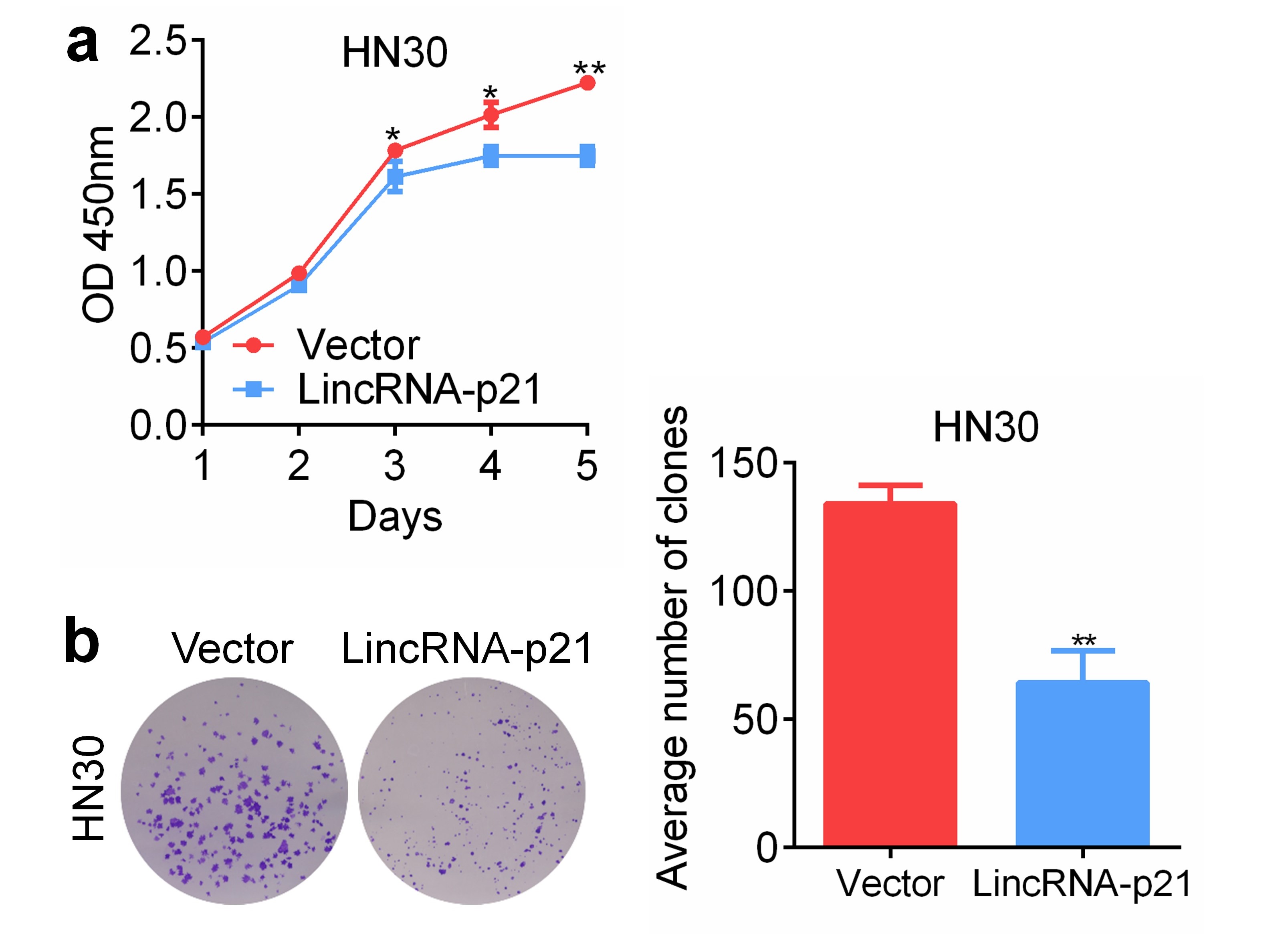


**Fig. S5** AKT and ERK1/2 signaling were analysed using western blot after si-lincRNA-p21 transfection for 48 h.


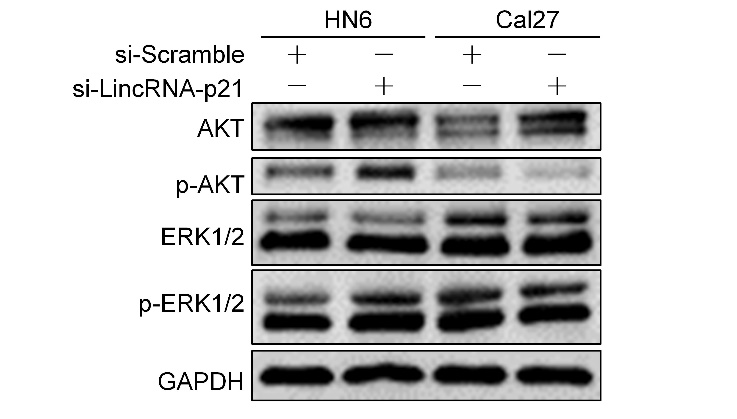


**Fig. S6** The transfection efficiency was confirmed in fresh xenograft tissue using qPCR.


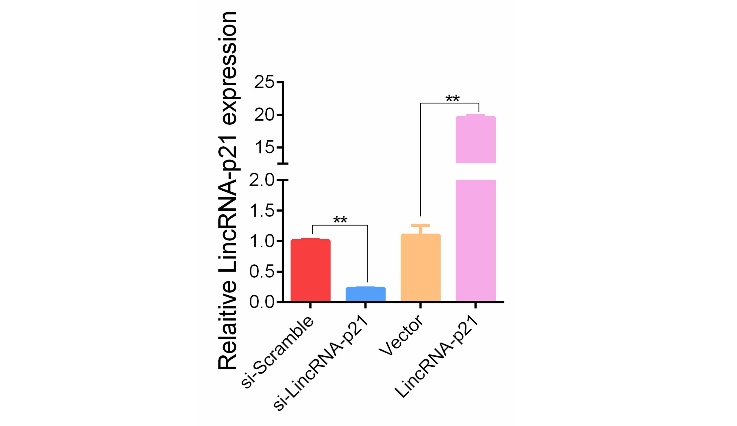


**Fig. S7** LincRNA-p21 regulates cell cycle and apoptosis related protein in HNSCC cells. (a) The cell cycle regulation related proteins were detected in HN6 and Cal27 cells after si-lincRNA-p21 for 48 h. (b) PARP, Caspase-3 and its active forms were detected in HN6 and Cal27 cells after si-lincRNA-p21 for 48 h.


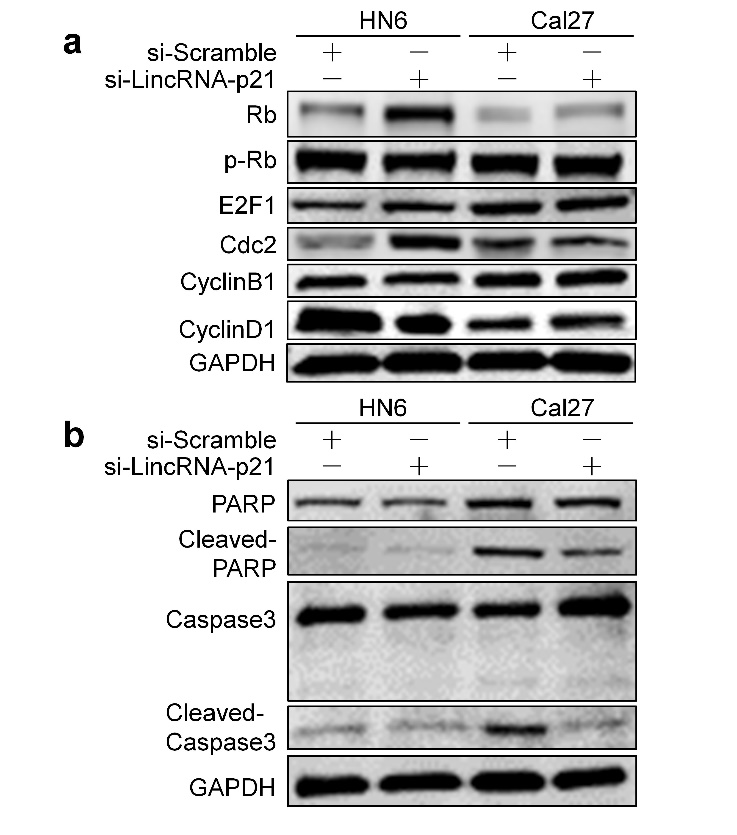


**Figure S8** Migration (a) and invasion (b) assays were performed with si-lincRNA-p21 or scrambled transfected HN6 and Cal27 cells using Transwell inserts.


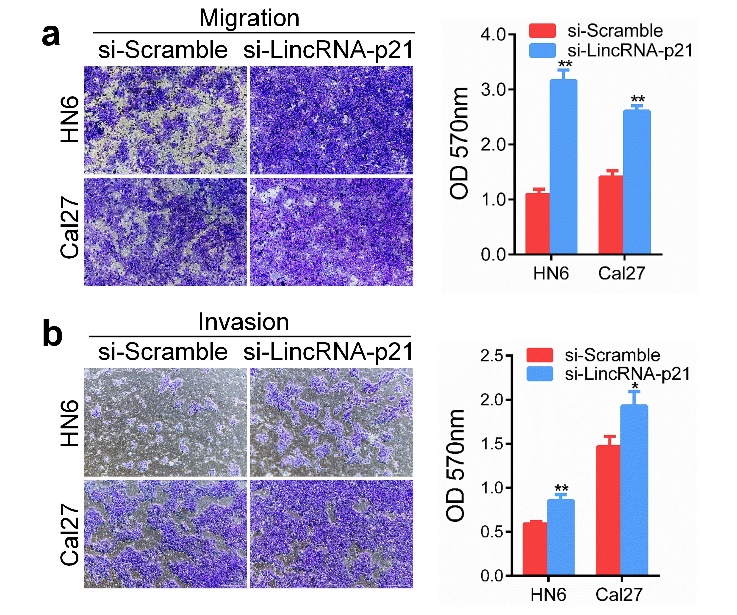


**Fig. S9** LincRNA-p21 reducing STAT3 expression is independent on ubiquitination degradation. Expression of STAT3 and Ubiquitin protein was detected after transfection for 48 h and then stimulation with 0.5 μM MG132 for 24h in HN6 and Cal27 cells.


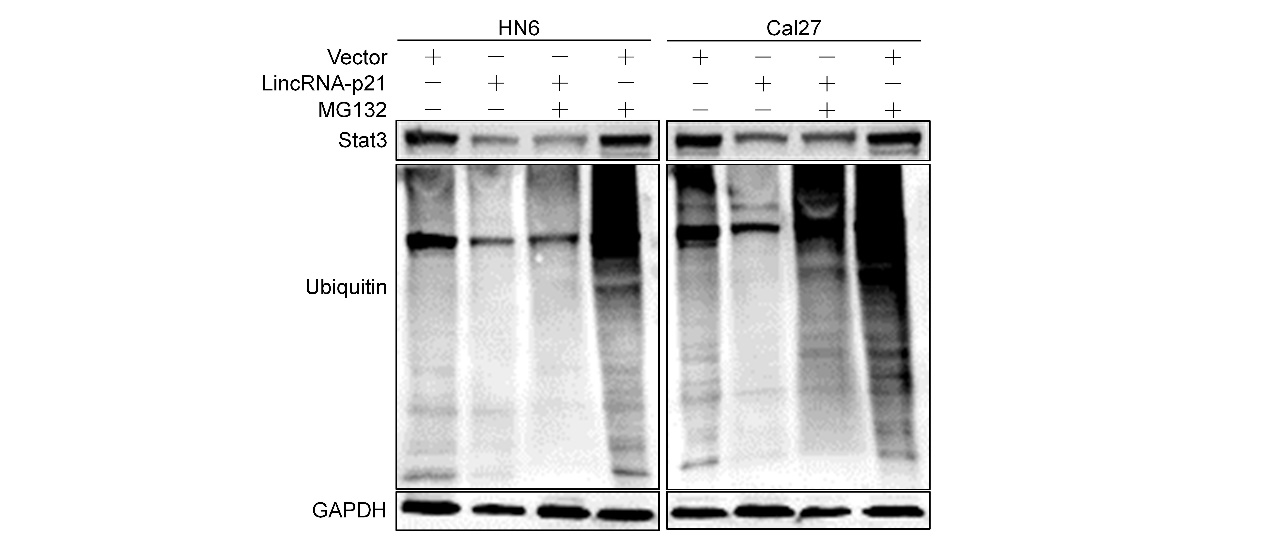


**Fig. S10** The staining score of p-STAT3 in in the xenograft tumour tissues.


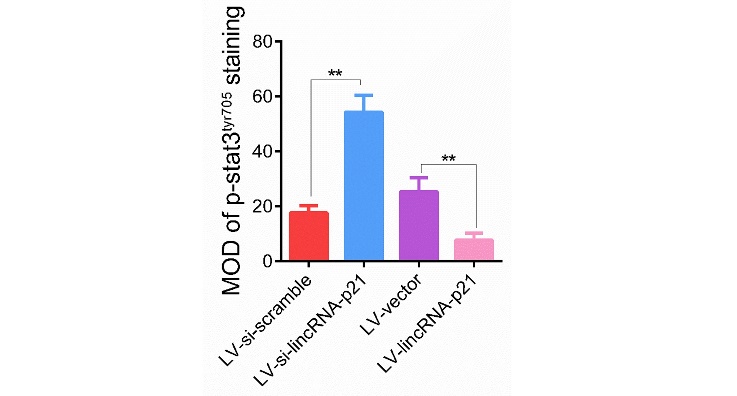


**Fig. S11** IC50 was calculated using cryptotanshinone (a STAT3 inhibitor) at indicated concentrations for 72 h in HN6 and Cal27 cells.


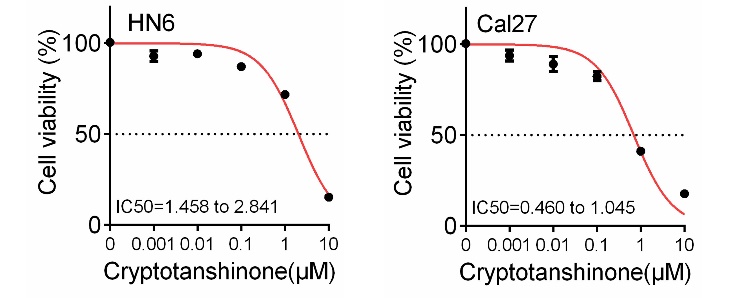

Supplement: Supplementary file 2 — Figure S1. The expression of lincRNA-p21 was detected by qPCR in breast cancer cells transfected with si-p53 for 24 h. Figure S2. (a) Transfection efficiency was detected after siRNA using qPCR in HN6 and Cal27 cells. (b)Transfection efficiency was detected after expression plasmid transfection using qPCR in HN6, HN30 and Cal27 cells. Figure S3. Cell viability (a) and colony formation ability (b) of HN6 and Cal27 cells after transfection with ASO-lincRNA-p21 or scrambled were determined using the CCK8 and colony formation assay. Figure S4. Cell viability (a) and colony formation ability (b) of HN30 cells after transfection with lincRNA-p21 or vector were detected using the CCK8 and colony formation assay. Figure S5. AKT and ERK1/2 signaling were analysed using western blot after si-lincRNA-p21 transfection for 48 h. Figure S6. The transfection efficiency was confirmed in fresh xenograft tissue using qPCR. Figure S7. LincRNA-p21 regulates cell cycle and apoptosis related protein in HNSCC cells. (a) The cell cycle regulation related proteins were detected in HN6 and Cal27 cells after si-lincRNA-p21 for 48 h. (b) PARP, Caspase-3 and its active forms were detected in HN6 and Cal27 cells after si-lincRNA-p21 for 48 h. Figure S8. Migration (a) and invasion (b) assays were performed with si-lincRNA-p21 or scrambled transfected HN6 and Cal27 cells using Transwell inserts. Figure S9. LincRNA-p21 reducing STAT3 expression is independent on ubiquitination degradation. Expression of STAT3 and Ubiquitin protein was detected after transfection for 48 h and then stimulation with 0.5 μM MG132 for 24 h in HN6 and Cal27 cells. Figure S10. The staining score of p-STAT3 in in the xenograft tumour tissues. Figure S11. IC50 was calculated using cryptotanshinone (a STAT3 inhibitor) at indicated concentrations for 72 h in HN6 and Cal27 cells. (DOCX 1296 kb) [file 12943_2019_993_MOESM2_ESM.docx]
